# Soft robotic constrictor for in vitro modeling of dynamic tissue compression

**DOI:** 10.1038/s41598-021-94769-2

**Published:** 2021-08-13

**Authors:** Jungwook Paek, Joseph W. Song, Ehsan Ban, Yuma Morimitsu, Chinedum O. Osuji, Vivek B. Shenoy, Dan Dongeun Huh

**Affiliations:** 1 Department of Bioengineering, University of Pennsylvania, Philadelphia, PA 19104 USA; 2 Department of Materials Science and Engineering, University of Pennsylvania, Philadelphia, PA 19104 USA; 3 Department of Chemical and Biomolecular Engineering, University of Pennsylvania, Philadelphia, PA 19104 USA; 4 Institute for Regenerative Medicine, Perelman School of Medicine, University of Pennsylvania, Philadelphia, PA 19104 USA; 5 NSF Science and Technology Center for Engineering Mechanobiology, University of Pennsylvania, Philadelphia, PA 19104 USA

**Keywords:** Lab-on-a-chip, Biomedical engineering, Tissue engineering

## Abstract

Here we present a microengineered soft-robotic in vitro platform developed by integrating a pneumatically regulated novel elastomeric actuator with primary culture of human cells. This system is capable of generating dynamic bending motion akin to the constriction of tubular organs that can exert controlled compressive forces on cultured living cells. Using this platform, we demonstrate cyclic compression of primary human endothelial cells, fibroblasts, and smooth muscle cells to show physiological changes in their morphology due to applied forces. Moreover, we present mechanically actuatable organotypic models to examine the effects of compressive forces on three-dimensional multicellular constructs designed to emulate complex tissues such as solid tumors and vascular networks. Our work provides a preliminary demonstration of how soft-robotics technology can be leveraged for in vitro modeling of complex physiological tissue microenvironment, and may enable the development of new research tools for mechanobiology and related areas.

## Introduction

**Figure 1:**
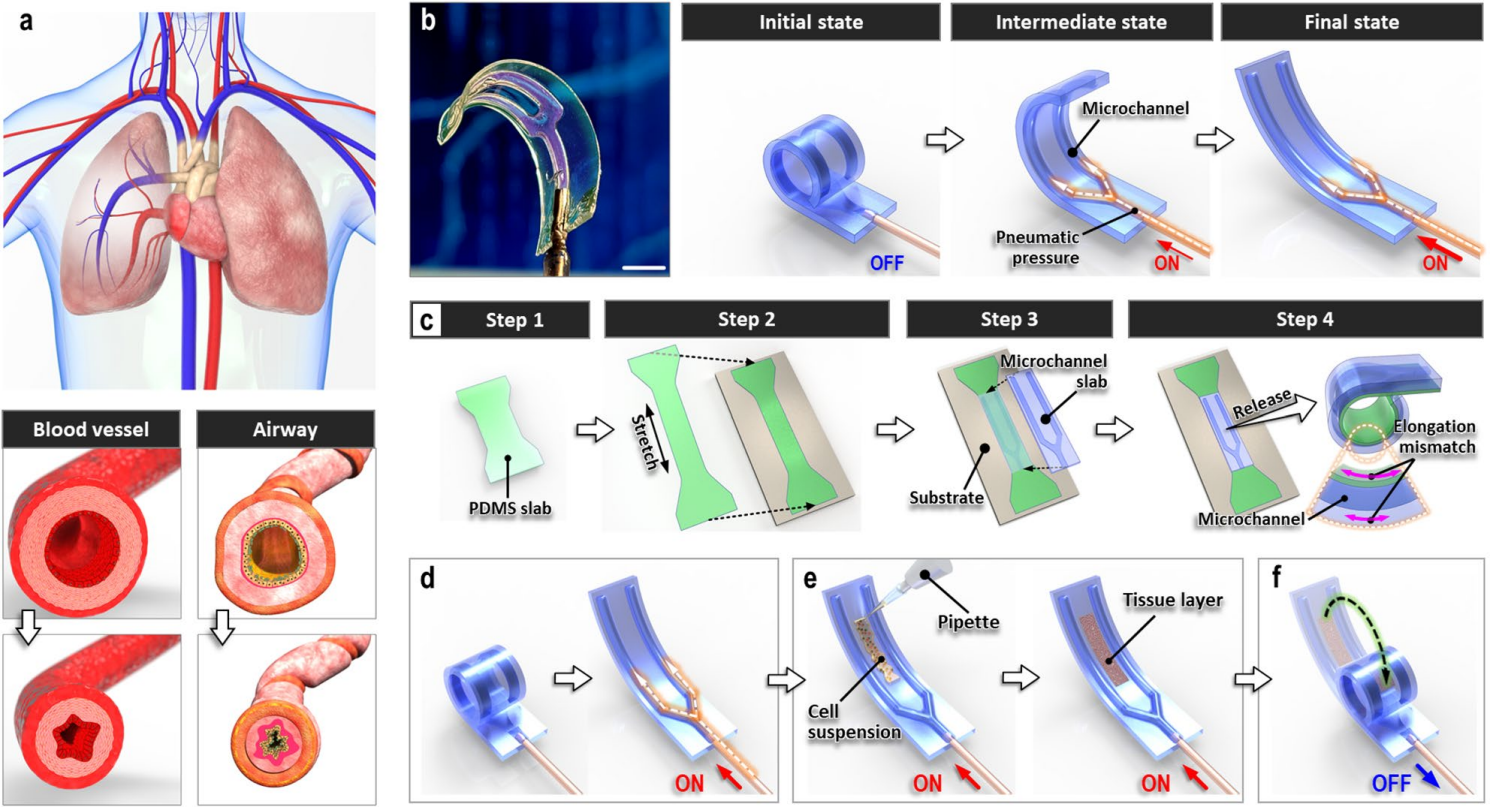
A soft-robotic constrictor for dynamic compressive mechanical loading of living tissue. (**a**) Circumferential tissue compression occurs during the constriction of tubular organs such as blood vessels and airways. (**b**) Normally coiled soft robotic cell culture platform and its pneumatically driven unrolling motion. Scale bar, 5 mm. (**c**) Fabrication of the soft robotic device through irreversible bonding of a thin stretched PDMS strip to a microchannel-containing PDMS slab (Steps 1, 2, and 3) and subsequent release of the bonded assembly (Step 4). (**d,e**) Sequential steps for establishing cell culture on the microengineered device. Red arrows labeled “ON” at the bottom of schematics represent application of pneumatic pressure to the microchannels. (**f**) Compression of cultured tissue layer through removal of air pressure from the microchannels (shown with a blue arrow labeled “OFF”) and the resultant bending of the culture substrate.

From blood vessels to the respiratory tract, various tubular structures in the body can undergo compressive deformation in the circumferential direction due to the contraction of muscle tissues in the outermost layer (Fig. 1a). By providing a mechanism to narrow the lumen and control fluid transport in a restrictive manner, this type of tissue deformation has been shown to play a crucial role in maintaining the homeostasis of many physiological systems^1–3^. When dysregulated, constriction of tubular organs can also be a hallmark of pathophysiological conditions such as obstructive pulmonary diseases^4–6^.

Our understanding about this unique feature of mechanically active organs has greatly improved over the last decades due in large part to significant progress in the study of muscle physiology. This body of work has elucidated molecular mechanisms responsible for feedback control of muscular contraction that occurs in response to a variety of endogenous and exogenous stimuli^7–10^. What remains to be further investigated, however, is whether and how other cellular constituents of these organs are influenced by mechanical forces that arise from the dynamic process of constriction. These questions have garnered great attention in recent years due to their implications in the study of complex disease processes. In asthma, for example, clinical evidence has shown that frequent bronchoconstriction in asthmatic lungs can lead to fibrotic remodeling of airway tissues in a manner independent of inflammation^11^. While research in mechanobiology has revealed the ability of various cell types to sense and react to naturally occurring physical forces, much of the work to date has focused primarily on cellular responses to stretch-induced tensile forces or fluid shear stress^12–14^.

Based on growing evidence suggesting the significant role of tissue compaction and compression in various developmental and physiological processes^15^, increasing efforts are being made to define the biological effects of compressive forces^16^. To conduct these types of studies in vitro, researchers have developed methods for simulating compressive tissue deformation that can be implemented in cell culture. For example, motorized linear actuators have been used to engineer devices capable of compressing fibroblasts in three-dimensional (3D) culture to model connective tissues under mechanical loading^17^. Similar approaches have been applied to investigating the effect of compressive forces on the development of tissue-engineered cartilage^18^. For the purposes of modeling the constriction of tubular tissues, however, existing techniques offer limited capacity to reproduce the dynamic process of mechanical loading in native organs. The main challenge has been to simulate the narrowing of the lumen and subject cultured cells to the resultant mechanical forces in a controllable manner. Attempts have been made to approximate this complex mode of tissue deformation using linear compression of cellular monolayers in two-dimensional (2D) settings^19–22^ but evidence also suggests the importance of 3D tubular architecture as a key determinant of stress fields generated by tissue distortion^23,24^. Therefore, for accurate in vitro modeling of constriction-induced mechanical forces and their biological effects, more advanced cell culture models are needed that can emulate 3D geometry of tubular structures and its dynamic changes during tissue deformation.

Here we describe an approach to address this need that exploits the unique capabilities of soft-robotics technology. Using microengineered elastomeric structures that can be controlled by computerized pneumatic actuation, we have developed a tissue-scale soft-robotic device capable of generating precisely regulated 3D bending motions. This system can be seamlessly integrated with cell culture and provides a platform to mimic circumferential compression and relaxation observed in tubular tissue structures in a more realistic manner. For proof-of-concept demonstration, we show dynamic cellular changes in primary culture of mechanosensitive human cells as a result of mechanical loading in the biomimetic soft robotic system. By incorporating organotypic 3D tissue constructs, we also demonstrate the utility of the microengineered soft robot for investigating the effect of compressive forces on (i) vasculogenic self-assembly and reorganization of blood vessels and (ii) the invasive potential of cancer cells.

## Results and discussion

### Device design and construction

Our soft-robot was designed as a normally bent/coiled elastomeric substrate that can be actuated to extend and unroll by controlled application of pneumatic pressure to a pair of embedded microchannels (Fig. 1b). Fabrication of this device begins with the preparation of a thin, dumbbell-shaped slab made out of poly(dimethylsiloxane) (PDMS) (Step 1 in Fig. 1c). This elastomeric layer is then stretched in the longitudinal direction and attached to a flat substrate (Step 2 in Fig. 1c). In the next step, another PDMS slab patterned with microchannel features is irreversibly bonded to the stretched PDMS layer affixed on the substrate (Step 3 in Fig. 1c). Release of the bonded device from the substrate results in immediate bending and rolling of the bi-layer structure due to a mechanical mismatch in residual stress between the unstretched and stretched PDMS layers (Step 4 in Fig. 1c).

To establish cell culture in this system, compressed air is injected into the microchannels embedded in the assembled device (Fig. 1d). During this process, the dead-ended channels sealed against a thin PDMS slab is inflated by pneumatic pressure, giving rise to forces that overcome residual stress to induce axial stretching and unrolling of the device—this working mechanism is the same as that of “party horn blowers”. Subsequently, cells of interest are deposited between the microchannels and allowed to attach and grow on the PDMS surface while keeping the device stretched (Fig. 1e). For mechanical loading, pressure is removed from the microchannels to induce the coiling of the device back into its original state (Fig. 1f), which approximates the circumferential contraction of a tubular tissue structure during the narrowing of its lumen. During this process, the bending motion exerts compressive stresses on the cultured cells initially grown on the stretched substrate. This actuation can be performed in an adjustable manner by changing key parameters, including the frequency and kinematics of pressure application, the level of applied pressure, and device dimensions. The stiffness of PDMS can also be altered as a means to change the dynamics of pneumatic actuation, which is readily achievable by varying the parameters of PDMS preparation and replica molding.

### Demonstration and characterization of soft-robotic actuation

**Figure 2:**
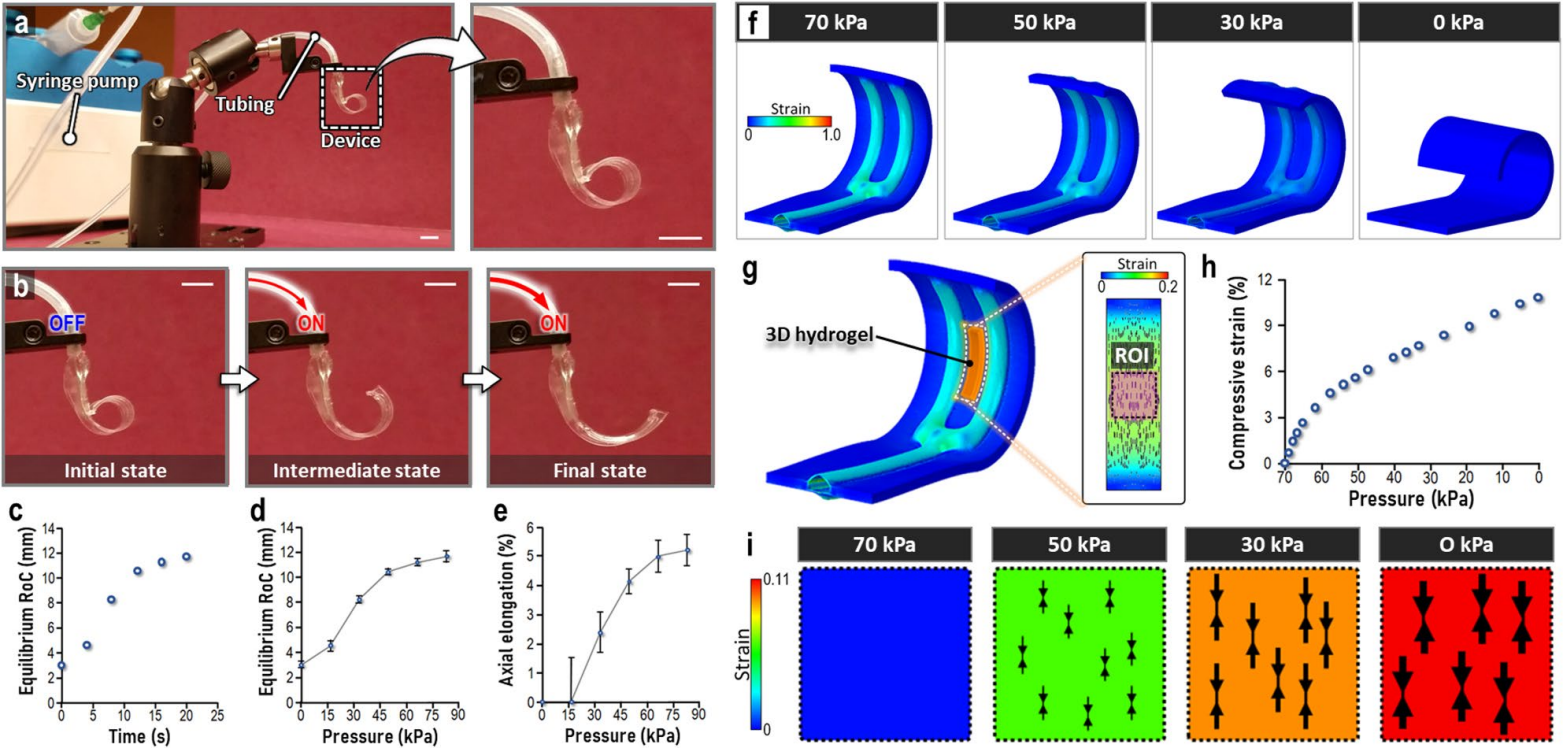
Pneumatic actuation of soft robotic constrictor. (**a**) Experimental setup for controlled pneumatical actuation of the soft robotic device. Scale bars, 5 mm. (**b**) Photographs of the uncoiling device due to applied air pressure. Scale bars, 5 mm. (**c**) Quantification of equilibrium RoC during the application of pneumatic pressure**.** Plots of equilibrium RoC (**d**) and axial elongation (**e**) at different levels of applied pneumatic pressure. (**f**) Finite element analysis-based computation prediction of substrate geometry and strain during pneumatic actuation of the soft robot. The heatmap shows maximum nominal principal strain. (**g**) Inclusion of 3D hydrogel for finite element modeling of 3D tissue. (**h,i**) In silico prediction of changes in mean compressive strain in the ROI with decreasing pneumatic pressure. The size of arrow pairs in (**i** is proportional to the level of compressive strain. The heatmap shows maximum nominal principal compressive strain. Data show mean ± SD with n = 3.

Following device construction, we first demonstrated and analyzed 3D motion of our soft-robot. In this study, we used a computer-controlled syringe pump connected to the assembled device as a source of compressed air needed for pneumatic actuation (Fig. 2a). Our system reacted to pressurization of the microchannels without significant delays by uncoiling the initially bent substrate in the longitudinal direction (Fig. 2b, Supplementary Movie 1). The structural deformation was quantitatively shown by the temporal increase in the radius of curvature (RoC) of the substrate during the application of pneumatic pressure (Fig. 2c). We found out that the equilibrium RoC at the completion of deformation increased proportionally with the level of pressure but began to plateau at approximately 12 mm when applied pressure reached 82.9 kPa (Fig. 2d). Pneumatic actuation also resulted in axial elongation of the device, which was measured to be roughly 5% when maximum deformation was achieved (Fig. 2e). It was noted that imperfect alignment of the microchannels during device assembly could result in a slight twist of the device in its relaxed state, but our analysis showed that this structural distortion did not produce significant torsional deformation of the device during pneumatic actuation (Fig. S1).

Successful demonstration of this soft-robotic actuation led us to ask whether the bending motion in our system generates stress fields that can be used for modeling physiological tissue distortion. To address this question, we developed a finite element model for in silico analysis of structural deformation in our device. As shown in Fig. 2f, when 70 kPa was applied to the device, the computational model predicted axially extended geometry of the elastomeric substrate, which was similar to the shape of our device during actuation. Release of pneumatic pressure resulted in a significant decrease in the level of strain in the microchannels, illustrating their volumetric shrinkage due to removal of compressed air (Fig. 2f). Concurrently, the device began to bend and coil in a manner consistent with our experimental observations (Fig. 2f, Supplementary Movie 2). The data indicated the greatest extent of deformation at 70 kPa, which was comparable to 82.9 kPa measured in our experiments. While further investigation is necessary, this difference might be attributed to pressure loss that occurs in our experimental setup, especially at interconnects and tubing between our device and the syringe pump.

Using this model, we then set out to conduct quantitative assessment of tissue mechanics in our device during pneumatic actuation. To this end, we incorporated a hydrogel construct between the microchannels in our computational representation of a maximally uncoiled device at 70 kPa to simulate a 3D tissue unit lining the lumen of a tubular structure in its relaxed state (Fig. 2g). Our analysis focused on examining deformation-induced strain fields generated in the middle of this tissue element defined as a region of interest (ROI) as applied pressure was decreased from 70 and 0 kPa to induce progressive bending of the culture substrate (Fig. 2g). As expected, our simulations showed no strain experienced by the relaxed construct at 70 kPa (Fig. 2h). When the device began to coil due to reduced pressure, however, the tissue element was put under compression as evidenced by the increase in the mean compressive strain (Fig. 2h,i). The strain continued to increase in a linear fashion with the decreasing level of pressure but when it reached approximately 60 kPa, we noticed a significant reduction in the rate of strain increase (Fig. 2h,i). At each applied pressure, the magnitude of the strain was maximized along the axial direction of the device (Fig. 2i), confirming that the strain fields were generated predominantly by pneumatically-driven bending of the tissue construct. When the compressive deformation of the device was completed at 0 kPa, the element in the ROI was subjected to a mean compressive strain of 11% (Fig. 2h). This maximum strain predicted by our model is similar to the physiological levels of circumferential strain experienced by airway tissues during breathing (12%) or blood vessels during vasoconstriction (10–20%)^25–27^.

Taken together, these results suggest that our system provides a controllable in vitro platform to approximate compressive tissue deformation and the resultant mechanical forces during the constriction of tubular tissue structures.

### Compressive mechanical loading of cell culture in the soft-robotic constrictor

**Figure 3:**
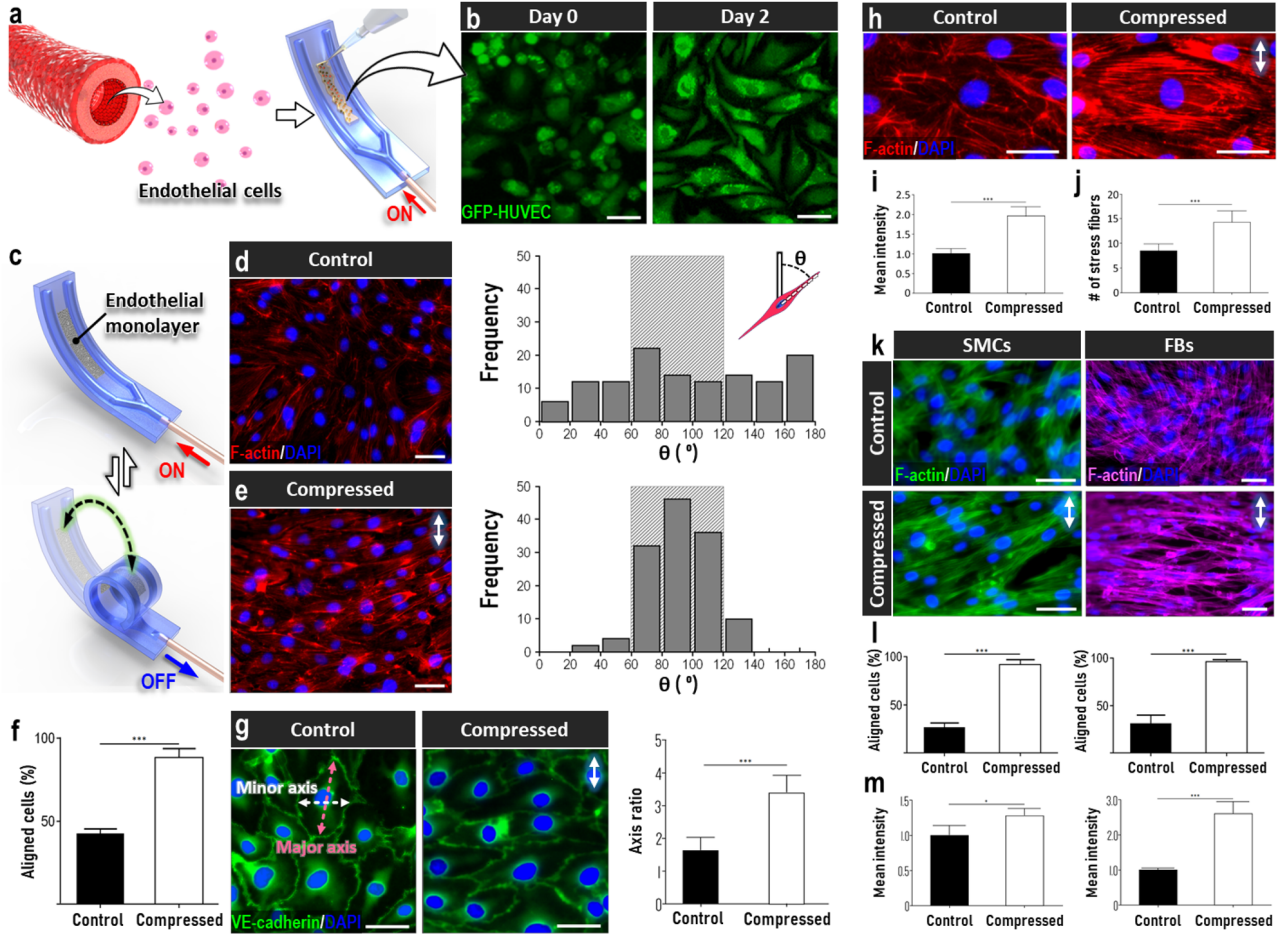
Responses of mechanosensitive cells to dynamic compression. (**a,b**) Monolayer culture of green fluorescence protein (GFP)-expressing HUVECs in the uncoiled device. Scale bar, 50 µm. (**c**) Cyclic pneumatic actuation of the endothelium-containing device. (**d–f**) Alignment of compressed endothelial cells due to applied compressive force as quantitatively assessed by the distribution of orientation angle (**d,e**) and the percentage of aligned cells (**f**). Scale bars, 50 µm. Any given cell was evaluated to be aligned when its orientation angle defined by the angle between its major axis and the vertical axis was between 60° and 120° (represented by the shaded boxes on the plots). (**g**) Compression-induced cell elongation quantified by the ratio of major to minor axes of cell body. Scale bars, 50 µm. (**h**) Fluorescence visualization of altered intracellular architecture of HUVECs due to compression. The architectural changes were quantified by the mean fluorescence intensity of F-actin staining (**i**) and the number of stress fibers (**j**). Scale bars, 50 µm. (**k**) Mechanosensitive responses of primary human smooth muscle cells (SMCs) and lung fibroblasts (FBs) as evidenced by their reorientation in the perpendicular direction to applied compressive force. Scale bar, 50 µm. (**l,m**) Quantification of cell alignment (**l**) and F-actin fluorescence intensity (**m**). Arrows in this figure show the direction of applied cyclic compression. ***P < 0.001, **P < 0.01, *P < 0.05. Data show mean ± SD with n = 3.

Having validated the design and operation of our system, we then established primary culture of human cells isolated from tubular organs and investigated their mechanosensitive responses in our device. First, we used primary human umbilical vein endothelial cells (HUVECs) as a model cell population and seeded them into the cell culture area between the actuation microchannels while the device remained uncoiled (Fig. 3a).

During culture, HUVECs continuously proliferated and formed a confluent monolayer over the entire culture surface within 2 days (Fig. 3b, Supplementary Fig. S2). For mechanical stimulation of the cultured cells, the device was actuated in a cyclic manner (Fig. 3c) to subject the endothelial monolayer to compressive forces reminiscent of those generated by the physiological process of vasoconstriction, which is known to occur at ~ 0.1 Hz^28^. In comparison to empty devices without cells, no significant changes were observed in the amount of pressure required for this dynamic compression (Fig. S3), indicating the negligible effects of the endothelial layer on device actuation. As shown in Figs. 3d,e, cyclic loading at 11% compressive strain for 5 consecutive days resulted in significant morphological changes in the endothelium. Most noticeable was that the vast majority of cells reoriented themselves in the direction perpendicular to applied strain. Our analysis indicated that over 88% of the cells exhibited the aligned morphology, and this percentage was substantially higher than that measured in the non-compressed control group (42%) (Fig. 3f).

Another characteristic phenotype of HUVECs in the compressed endothelium was cytoplasmic elongation, which was clearly visible in immunostaining of the endothelial adherens junction component, VE-cadherin (Compressed, Fig. 3g). This feature was not observed in the absence of compression, in which case the endothelial cells retained their polygonal, cobblestone-shaped morphology (Control, Fig. 3g). This difference was further demonstrated by the greater ratio of major to minor axes of the cells in the compressed group (Fig. 3g). In addition to these shape changes, mechanical loading in our device also altered the intracellular architecture of HUVECs as illustrated by the formation of well-defined actin bundles in the cytoplasm of the compressed cells (Fig. 3h). This observation was verified quantitatively by significant increases in the mean fluorescence intensity of overall actin staining (Fig. 3i) and the number of cytoplasmic stress fibers (Fig. 3j). In addition to these cellular alterations, our data also showed that the ability of our system to generate controlled structural deformation remained unchanged after 5 days of continuous dynamic compression (Fig. S4), demonstrating the stability of mechanical actuation in our system for prolonged periods.

The functional capacity of our system for dynamic mechanical loading of cell culture was further demonstrated by incorporating other types of mechanosensitive cells, such as primary human vascular smooth muscle cells and lung fibroblasts. Compression of these cells using the same regimen led to similar directional alignment and increased polymerization of actin cytoskeleton when compared to their non-compressed counterparts (Fig. 3k–m). Importantly, our data obtained from these three distinct cell types match previous in vivo and in vitro findings^29–31^, and demonstrate the capability of our device to reproduce physiologically relevant compressive forces and their potential to induce morphological alternations and remodeling at the cellular and tissue levels.

### Soft robotic compression of 3D human tissues

**Figure 4:**
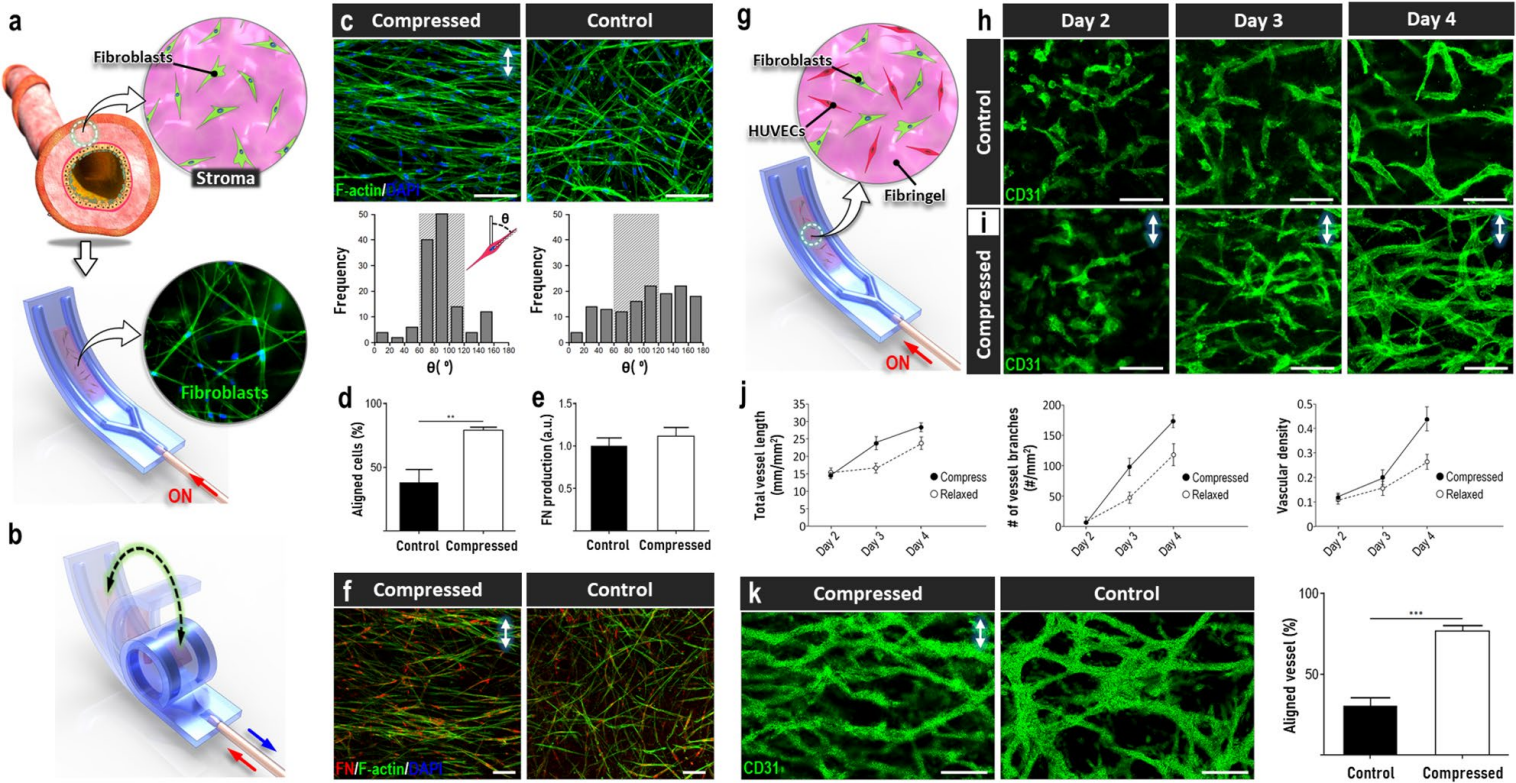
Dynamic soft robotic compression of cells and tissues in 3D culture. (**a,b**) In vitro modeling of stromal tissue in the lung using a fibroblast-laden collagen hydrogel construct formed in the soft robotic device. (**c,d**) Alignment of cultured fibroblasts due to cyclic compression. Any given cell was evaluated to be aligned when its orientation angle defined by the angle between its major axis and the vertical axis was between 60° and 120°, (represented by the shaded boxes on the plots). Scale bars, 100 µm. (**e,f**) The production of FN by fibroblasts was not influenced by compressive mechanical loading. Scale bars, 100 µm. (**g**) Co-culture of primary human lung fibroblasts and HUVECs for in vitro modeling of vasculogenesis. (**h,i**) Self-assembly of 3D vascular networks over a period of 6 days in the absence (**h**) or presence of compression (**i**). Green shows CD31 staining. Scale bars, 50 µm. (**j**) Quantification of total vessel length, the number of vascular junctions, and vascular density. (**k**) Formation of de novo vessels aligned perpendicularly to applied compression. Scale bars, 50 µm. Arrows in this figure indicate force direction. ***P < 0.001, **P < 0.01, *P < 0.05. Data show mean ± SD with n = 3.

In the next phase of our study, building upon the proof-of-concept demonstration of soft robotic mechanical loading in 2D cell culture, we explored the feasibility of modeling 3D human tissues under compression. For this investigation, we first constructed a simple model of stromal tissue in the human respiratory tract by generating primary human lung fibroblast-laden type I collagen hydrogel in our robotic platform (Fig. 4a). The engineered hydrogel construct was then subjected to cyclic compression to emulate expiration- or disease-induced circumferential compression of airway walls in vivo (Fig. 4b)^32^.

In response to sustained mechanical loading for 5 days at a physiological strain of 11%^25^ and at normal breathing frequency (0.25 Hz)^33^, the stromal tissue underwent noticeable microarchitectural changes characterized by the perpendicular alignment of the embedded fibroblasts relative to the direction of applied force (Compressed in Fig. 4c,d). This compression-induced cellular reorganization, which is known as a mechanoresponsive phenotype of lung fibroblasts^34^, did not occur to any measurable extent in static culture in which the fibroblasts remained randomly oriented (Control in Fig. 4c,d). Considering the primary role of fibroblasts in the regulation of extracellular matrix (ECM) in the connective tissue, we also assessed whether mechanical stimulation affected their ECM environment by immunostaining fibronectin (FN) as a representative matrix protein. As shown in Fig. 4e,f, our analysis did not show any significant changes in the production and deposition of FN in the hydrogel scaffold compared to the control group. This result, which is in contrast to the drastic morphological rearrangement due to compression, might be indicative of functional capacity of fibroblasts to maintain ECM homeostasis in the face of dynamically applied physiological mechanical forces, which has previously been shown by in vitro studies^35^.

Inspired by extensive evidence showing the essential role of mechanical forces in embryogenesis^15^, we also set out to demonstrate the use of our microengineered soft robotic system for studying the effect of compressive deformation on tissue and organ development. This study was conducted by modeling the process of vasculogenesis in our device. Despite the fact that the development of blood vessels during embryogenesis has been a topic of intense research attention^36^, it remains poorly understood how this process is affected by dynamic mechanical forces, especially those generated by tissue compaction and contraction commonly observed in a developing embryo^37^. A key challenge for investigating this question has been the limited capacity of conventional in vitro techniques to model the complex process of tissue vascularization in a dynamic biomechanical environment.

To tackle this challenge, we first established 3D co-culture of primary human vascular endothelial cells and fibroblasts in our device to mimic de novo formation of 3D vascular networks (Fig. 4g). During culture under static conditions, the endothelial cells in fibrin gel formed intercellular connections and assembled themselves into a 3D network of interconnected tubular structures over a period of 4–6 days (Fig. 4h). When the device was pneumatically actuated to generate cyclic compression, however, this spontaneous process of vessel formation and assembly appeared to take place in an accelerated manner (Fig. 4i). This observation was supported by quantification of key vascular features over the course of cell culture, which showed significantly larger vessel length, branch numbers, and vascular density in the compressed group at given time points (Fig. 4j). Another notable finding was that the endothelial cells in the compressing hydrogel scaffold showed the tendency to reorient themselves perpendicularly to applied loading. As a result, the self-assembled vasculature developing under this dynamic condition displayed more aligned morphology (Fig. 4k).

These data suggest that naturally occurring compressive mechanical stimulation may influence the development of blood vessels, potentially contributing to morphological maturation of the vasculature. Indeed, the results of our morphometric analysis are consistent with the findings of previous studies showing the potential of mechanical strain to promote endothelial cell growth and blood vessel formation^38^. It should be noted, however, that our work provides the first in vitro demonstration of enhanced vascular development due to dynamic tissue compression. The physiological relevance of compression-induced vascular alignment described above remains to be further investigated but this observation may have implications in understanding the development and organization of vascular tissues in various mechanically active tubular organs (e.g., lung, gut) that experience cyclic structural deformation during embryonic and fetal development^39^.

### Probing the effects of dynamic mechanical forces on cancer invasion

**Figure 5:**
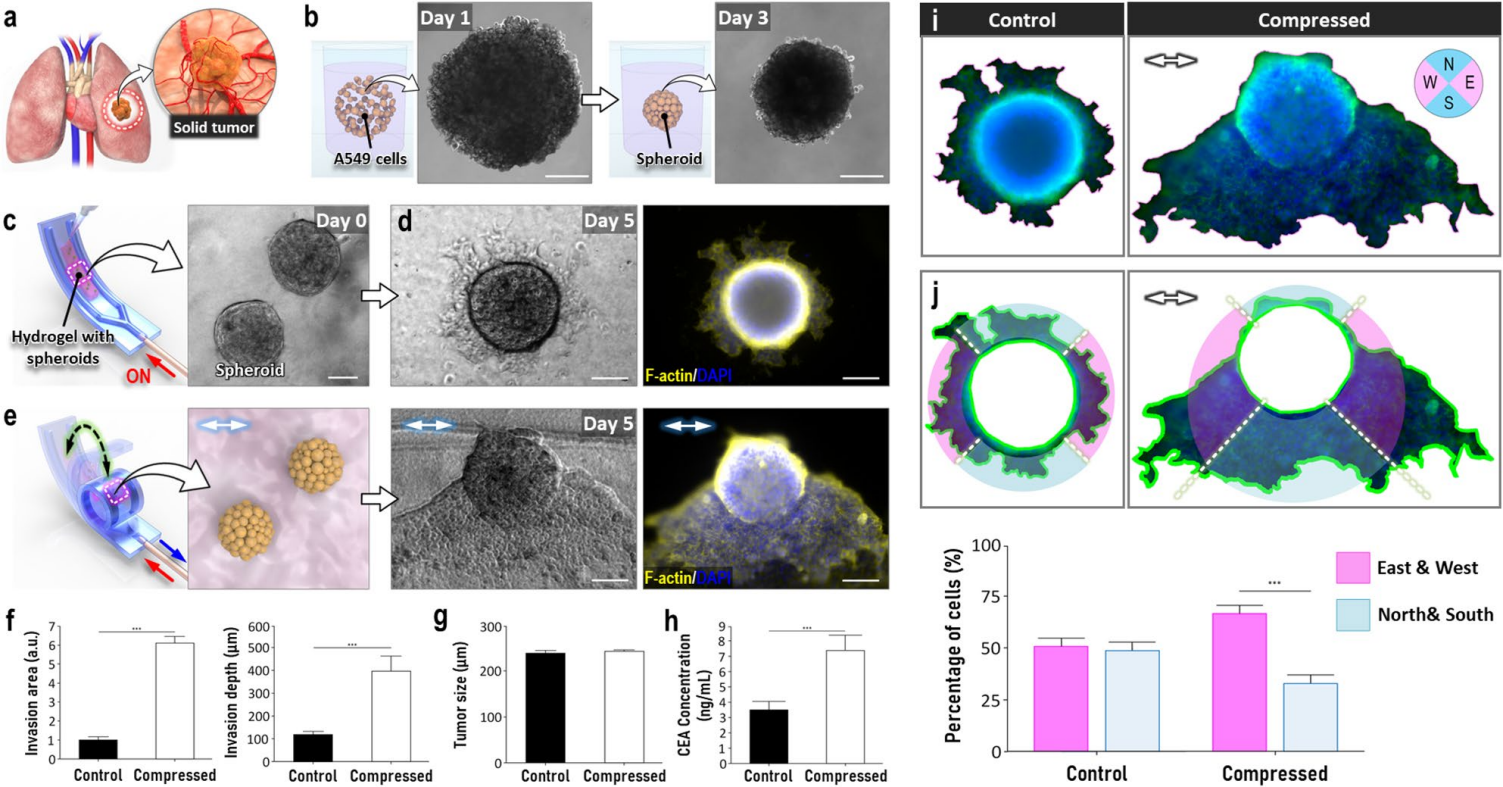
In vitro modeling of solid tumor in the soft robotic constrictor. (**a,b**) A549 spheroids formed in low-attachment wells are used to model malignant tumors in the lung. Scale bars, 100 µm. (**c**) Micrograph of tumor spheroids embedded in type I collagen hydrogel in the device. Scale bar, 100 µm. (**d**) Phase contrast (left) and fluorescence (right) images of lung tumor after 5 days of static culture. Scale bars, 50 µm. (**e**) Micrographs of dynamically compressed lung tumor at Day 5. Scale bars, 100 µm. (**f**) Quantification of invasion area and depth. (**g**) Comparison of tumor size in the presence or absence of compression. (**h**) ELISA-based quantification of CEA secreted by lung tumors. (**i**) Delineation of the outline of tumor and invading cancer cells using fluorescence micrographs shown in (**d,e**) E, W, S, and N in the circular direction map represent east, west, south, and north, respectively. (**j**) Radial segmentation of tumor regions and quantification of directional cancer cell migration. Arrows in this figure show the direction of applied compressive force. ***P < 0.001, **P < 0.01, *P < 0.05. Data show mean ± SD with n = 3.

Finally, we designed a preliminary study to investigate whether our device could be used as a platform for in vitro modeling of human disease. Motivated by ongoing research efforts to understand the biomechanical basis of cancer development and progression^40^, this work focused on modeling malignant tumors in the human lung (Fig. 5a), with the specific goal of examining how compressive tissue deformation due to the dynamic activity of the respiratory system affects the invasive behavior of cancer cells.

As a first step to construct this model, A549 human lung adenocarcinoma cells were cultured in low-attachment wells to form cancer spheroids reminiscent of solid tumors in the lung (Fig. 5b). Once collected from the wells, the spheroids were mixed with type I collagen precursor solution and introduced into our device to generate 3D tumor constructs (Fig. 5c). In static culture without pneumatic actuation, the A549 spheroids were stably maintained in the hydrogel scaffold without any measurable loss of tissue integrity but over a period of 5 days, we observed outgrowth of cancer cells from the spheroids into the surrounding environment (Fig. 5d). Interestingly, when tumor constructs were grown in devices put under compression, this invasive phenotype of the spheroids became more pronounced (Fig. 5e) as demonstrated by more than six and fourfold increases in the area and depth of cancer cell invasion, respectively (Fig. 5f). There was no statistical difference in the tumor size between the two groups after the same duration of culture (Fig. 5g) but greater numbers of outgrowing and invading cells were seen in the compressed constructs (Fig. 5e). This result is suggestive of increased total cell number in the mechanically loaded tumors, which is consistent with previous findings about enhanced proliferation of cancer cells in a compressive tumor microenvironment generated by solid pressure^41^. This observation was further supported by significantly higher concentrations of cancer cell marker, carcinoembryonic antigen (CEA), in conditioned media sampled from the compressed devices (Fig. 5h).

Our investigation also revealed the directional outgrowth of cancer cells under compression. In the control group maintained under static conditions, the cells migrated out of the spheroids to more or less the same extent in all directions (Control in Fig. 5i). In contrast, cellular invasion in the compressed model clearly showed directionality with the majority of cells moving preferentially in the east, south, and west directions of the spheroids (Compressed in Fig. 5i). In attempting to quantitatively analyze this difference, we found out that the direction of applied compression may influence the spatial distribution of outgrowing cells. When the tumors and their surrounding environment were radially segmented into four equal regions of analysis, the percentage of cells migrating in the two opposing regions along the axis of compression was significantly higher than that in the other two segments (Fig. 5j). Based on this result, we speculate that many of the invading cancer cells in this model may have the ability to sense the direction of applied forces and use this as a spatial cue to guide their migration into the surrounding matrix. It should be noted, however, that a fraction of cells still migrate in the direction perpendicular to applied compression (preferentially in the south direction), which may suggest cellular variability or spatial anisotropy of our model that needs to be further investigated.

Although preliminary, these data show the possibility of using our device to study invasive properties of malignant tumors under the influence of physiologically relevant biophysical forces that occur in mechanically active organs. Obviously, for more accurate modeling of the disease phenotype, further development is necessary to reproduce the cellular heterogeneity of the native tumor microenvironment and complex biological crosstalk between multiple cell types. Considering that the engineered tumor tissues in this device are easily accessible and can readily be manipulated, our system may also enable more in-depth studies of malignant disease processes beyond the morphometric analysis of cancer cell invasion.

## Conclusion

The work described here provides a good example of how soft-robotics can be combined with conventional 2D and 3D cell cultures to address the experimental burden of modeling cellular and tissue responses to physiological mechanical forces in vitro. By exploiting the flexibility of PDMS, we developed a pneumatically addressable cell culture substrate with simple operation that can be used for simulating tissue deformation during the constriction of tubular organ units. As demonstrated in this work, our soft-robotic constrictor enables dynamic compression of cells in 2D and 3D culture to investigate their mechanoresponsive properties in a controlled environment.

While the presented data show the potential of our device as a novel research platform, work remains to be done to further improve its capabilities and explore new opportunities. For example, the maximum compressive strain attainable in the current device is 11%, which may limit the application of our system to modeling pathophysiological tissue deformation known to generate significantly higher strain levels. In case of obstructive lung diseases, for instance, studies have estimated 25–30% strain during excessive constriction of the respiratory tract^5,25,33^. Tackling this limitation will require modification and optimization of device design and/or PDMS stiffness, which may be facilitated by predictive in silico analysis using our finite element model. Although our study focused on compressive mode of tissue deformation, it is possible to use the same device for mimicking tensile force-induced deformation if methods can be devised to plate and grow cells initially in the coiled substrate before actuation. This new capability will greatly enhance the versatility of our platform and allow for broader application of the system. Moving beyond proof-of-concept demonstrations presented here, future studies should also investigate the feasibility of integrating more realistic and physiological tissue constructs and organotypic cultures to increase the biological complexity of the dynamic cell culture environment that can be engineered in our device.

With further development, we believe that soft-robotic engineering of cell culture demonstrated in this study will contribute to advancing the state-of-the-art for in vitro modeling of complex physiological systems for biomedical and pharmaceutical applications.

## Methods/experimental

### Device fabrication

We used standard soft lithography techniques to fabricate a poly(dimethylsiloxane) (PDMS) blank slab and microfabricated channel slab patterned with recessed features of microscale pneumatic channels illustrated in Fig. 1c. The dimensions of the device and cell-culture surface were 10 mm (width) × 30 mm (length) × 0.66 mm (height) and 2 mm (width) × 5.5 mm (length), respectively (Fig. S5). The cross-sectional dimension of the microchannels was 2 mm (width) × 400 µm (height) (Inset of Fig. S5). The stretchable PDMS blank slab was produced by molding PDMS into a dumbbell-shaped thin film with a thickness of 125 µm (Fig. S6). For fabrication of these components, PDMS (Sylgard 184, Dow Corning) base was mixed with a curing agent at a weight ratio of 10:1 (base:curing agent) and poured onto 3D-printed masters (ProtoLab). After degassing, PDMS was fully cured in an oven maintained at 65 °C and then, removed from the molds. PDMS slabs prepared by this procedure had a Young’s modulus of 1.9 ± 0.01 MPa. For device assembly, the dumbbell-shaped PDMS blank slab was stretched by 200% and attached to a plastic substrate using double-stick tape (3 M). Afterwards, a thin layer of PDMS was prepared on the stretched PDMS blank slab and plastic substrate by spin-coating at 500 rpm for 1 min and then, semi-cured in an oven maintained at 65 °C for 15 min. Following this step, another PDMS slab patterned with microchannel features was bonded to the stretched PDMS blank slab on the plastic substrate using the thin layer of semi-cured PDMS as an adhesive. Then, the assembled device was baked at 65 °C to fully cure the PDMS adhesive layer. Upon the completion of baking process, we manually detached the assembled device from the double-stick tape and plastic substrate. Finally, we connected the open-end of the pneumatic channel of the device with a blunt needle, which served as an airflow access port for pneumatic actuation.

### Finite element model of pneumatically driven compression

The deformation of pneumatic actuator was modeled using the finite element method. Initially, a planar geometric model of the actuator was generated. This model was meshed using the finite element package Abaqus (Simulia). A portion of the mesh was then transformed into a cylinder-like shape to mimic the shape of the experimentally fabricated device. The mesh consisted of about 70,000 tetrahedral second-order elements. PDMS was modeled as a nearly incompressible neo-Hookean material using the strain energy density function

1 $$W ={C}_{1}\left(\overline{I }-3\right)+{D}^{-1}{\left(J-1\right)}^{2},$$

where $\overline{I }$ is the first invariant of the isochoric part of the right Cauchy-Greene deformation tensor $\overline{\mathbf{C} }={J}^{-2/3} \mathbf{C}={{J}^{-2/3}\mathbf{F}}^{\mathrm{T}}\mathbf{F}$. Here, $\mathbf{F}$ is the deformation gradient tensor defined as ${\nabla }_{{\varvec{X}}}{\varvec{x}}$, where ${\varvec{x}}$ and ${\varvec{X}}$ denote the positions of material points after and before deformation, respectively^42^. $J$ is defined as $\mathrm{det}(\mathbf{F})$. ${C}_{1}$ was chosen as 700 kPa^43^ and $D\ll$ 1 was used to model near incompressible deformation. The deformation of collagen in compression was modeled as linear by an elastic modulus of 1.15 kPa and vanishing Poisson’s ratio^44^. Symmetric boundary conditions were employed. The actuator was clamped at the air inlet and fixed vertically at the cross-section near the inlet. Because collagen was polymerized at the pressurized state of the actuator, its compressive strain in linear deformation was calculated based on a transformed deformation gradient $\mathbf{F}{\mathbf{F}}_{\mathrm{pr}}^{-1}$. ${\mathbf{F}}_{\mathrm{pr}}$ is the deformation gradient when the actuator is pressurized.

### Cell culture

To demonstrate the proof-of-principle for soft-robotic modeling of mechanically dynamic cellular-environment, we used primary human umbilical vein endothelial cells (HUVECs), primary normal human lung fibroblasts (NHLFs), and primary human uterine smooth muscle cells (HUtSMCs). HUVECs, NHLFs, and HUtSMCs were cultured in 25-cm^2^ flasks according to the manufacture’s protocols using endothelial cell growth medium (EGM)-2 (CC-3162, Lonza), fibroblast growth medium (FGM)-2 media (CC-3132, Lonza), and smooth muscle cell growth medium (PCS-100-042, ATTC), respectively. Cells between passage 3 and 5 were used for device culture.

For 2D cell-culture (Fig. 3), the fully assembled device was sterilized by exposing it to ultraviolet (UV) light (Electro-lite ELC-500) for at least 30 min. Subsequently, the cell-culture surface on the device was introduced and incubated with a fibronectin solution (0.1 mg/ml in phosphate-buffered saline (PBS)) (Corning-356008) to generate ECM coating on the surface for cell attachment. After 2-h incubation, the channel was washed with PBS, filled with culture medium, and incubated in a cell culture incubator at 37 °C and 5% CO_2_ for 2 h. As the first step of cell seeding, trypsinized cells were suspended in their growth medium at a concentration of 1 × 10^6^ cells/ml, and the cell suspension was introduced on PDMS surface between two microchannels of our device that was kept in an uncoiled state with the application of pneumatic pressure. Immediately after seeding, the whole device was incubated in a cell-culture incubator at 37 °C and 5% CO_2_ which allows the seeded cells to settle to the cell-culture surface and attach for future experiments.

The process of 3D cell-culture shown in Figs. 4 and 5 started out with the creation of functional coating for enhanced adhesion of ECM hydrogel to PDMS cell-culture surface in our microengineered device. To this end, the cell-culture surface of sterilized device was treated with a buffer solution containing 0.2 mg/ml of sulfosuccinimidyl 6-(4′-azido-2′-nitrophenylamino) hexanoate (Sulfo-SANPAH, 13414, Covachem) and irradiated with 365 nm UV light for 10 min, followed by rinsing steps with DPBS (356008, Corning). To generate NHLF-laden collagen hydrogel constructs in the device, the cells were mixed with a 2 mg/ml collagen type I solution (#354236, Corning, USA) at a final density of 0.5 × 10^6^ cells/ml. 100 µl of the mixture solution was then placed onto the functionally coated cell-culture surface of our uncoiled device. Afterwards, the device was incubated at 37 °C and 5% CO_2_ to induce gelation. Following 30-min incubation, culture medium was added to the solidified collagen hydrogel containing NHLFs for further investigations.

### Construction of in vitro vascular model

To form 3D microvascular beds in our microengineered device, NHLFs and HUVECs were suspended each at 5 × 10^6^ cells/ml in a mixture of fibrinogen (10 mg/ml in PBS) and thrombin (1 U/ml in PBS), and then introduced to the functionally coated cell-culture surface in our device. Subsequently, the seeded device was placed in a 37 °C incubator with 5% CO2 for 30 min, during which fibrinogen was enzymatically converted to fibrin. Upon completion of the gelation step, (EGM)-2 medium was added to the cell-laden fibrin hydrogel construct.

### Production of tumor model

Tumor spheroids were generated by using a A549 human lung adenocarcinoma cell line (CL-185, ATTC). A549 cells were cultured in ultra-low attachment 96-well plates (4515, Corning) with their growth media (30-2004, ATTC). For each well, 100 µl of cell suspension containing 1000 A549 cells were added, and the cells were allowed to settle and form a loose aggregate. After overnight culture, 100 µl of growth medium was pipetted into each well. A549 spheroids generated over a period of 3 days were harvested for use in our devices. Then, the A549 spheroids were mixed with a 2 mg/ml collagen type I solution (#354236, Corning, USA) and introduced on the functionally coated cell-culture surface in our device to create a 3D lung tumor construct. After gelation of the collagen hydrogel scaffold, culture medium was added to the device which was subsequently incubated at 37 °C and 5% CO_2_ for studying on effects of mechanical loading to metastatic behaviors of the cancer cells.

### Immunostaining

For immunostaining, cells in our devices were fixed in 4% paraformaldehyde for 15 min at RT, washed with DPBS, and permeabilized with 0.25% Triton X-100 for 10 min. Subsequently, a blocking step was performed using 3% bovine serum albumin (BSA) overnight at 4 °C. The cells were then incubated overnight at 4 °C with primary antibodies. For imaging endothelial junctions and self-assembled vessels, we used rabbit polyclonal anti-VE-cadherin (ab33168, 1:100, Abcam) and rabbit polyclonal anti-CD31 (ab28364, 1:100, Abcam) primary antibodies. Analysis of fibronectin deposition in the airway stromal tissue model was achieved by using mouse monoclonal anti-fibronectin (sc-59826, 1:150, Santa Cruz Biotechnology) antibody. After incubation with primary antibody, the cells were washed twice with PBS and incubated for 1 h at RT with secondary antibody (ab150077, Abcam; A-32723, ThermoFisher). Visualization of actin cytoskeleton was achieved by treating the cells with Alexa Fluor 594 conjugated phalloidin (Life Technologies) for 30 min. We also used Hoechst (33342, ThermoFisher) for nuclear staining. Fluorescence images of the cells were captured by an inverted epi-fluorescence microscope equipped with a confocal laser scanning module (LSM 800; Carl Zeiss, Germany). The obtained images were processed using the ZEN software (Zeiss, Germany).

### Quantification

The orientation angle of cell body was analyzed in ImageJ by identifying the major axis of the cell body with one-segment line ROIs and measuring the angle to the verticalaxis (corresponding to the direction of compression). For analyzing vascular alignment, we first extracted a vessel segment between two adjacent vascular branches and measured its angle to the direction of mechanical loading using the Zen software (Zeiss Microscopy). Based on the measured angles, cells and vascular segments were divided into two groups, each of which represented 60°–120° directionality (perpendicular to the direction of compressive force) and random directionality, respectively.

For quantitative metrics for assessment of compression-induced alterations in intracellular architecture, we calculated the mean fluorescence intensity of F-actin staining and the average number of stress fibers using the Zen software and ImageJ. We also used the Vessel J to quantify and evaluate the rate of vessel formation during the vasculogenic process shown in Fig. 4h.

Lastly, immunofluorescence images used in our analysis were taken from three independent experiments.

### Measurement of Young’s modulus for PDMS

To evaluate Young’s modulus of PDMS slabs used for device fabrication, their tensile properties were measured using ARES-G2 (TA Instruments, Inc., DE, USA). The measurement was carried out using three rectangular PDMS slabs (30 mm × 10 mm × 2.5 mm) at room temperature with a crosshead speed of 1 mm/min. The acquired tensile property data were used to calculate Young’s modulus as shown in Fig. S7.

### Statistical analysis

Statistical significance of the obtained data was evaluated by a two-tailed t-test. Data were presented as mean ± SD.

## Supplementary Information

Supplementary Figures.

Supplementary Video 1.

Supplementary Video 2.
