# Supplementary material for: Soft robotic constrictor for in vitro modeling of dynamic tissue compression
Source: Sci Rep. 2021 Aug 13;11:16478. doi: 10.1038/s41598-021-94769-2 (PMC8363742; doi:10.1038/s41598-021-94769-2)
Supplement: Supplementary file 1 — Supplementary Figures. [file 41598_2021_94769_MOESM1_ESM.pdf]

Supplementary Information

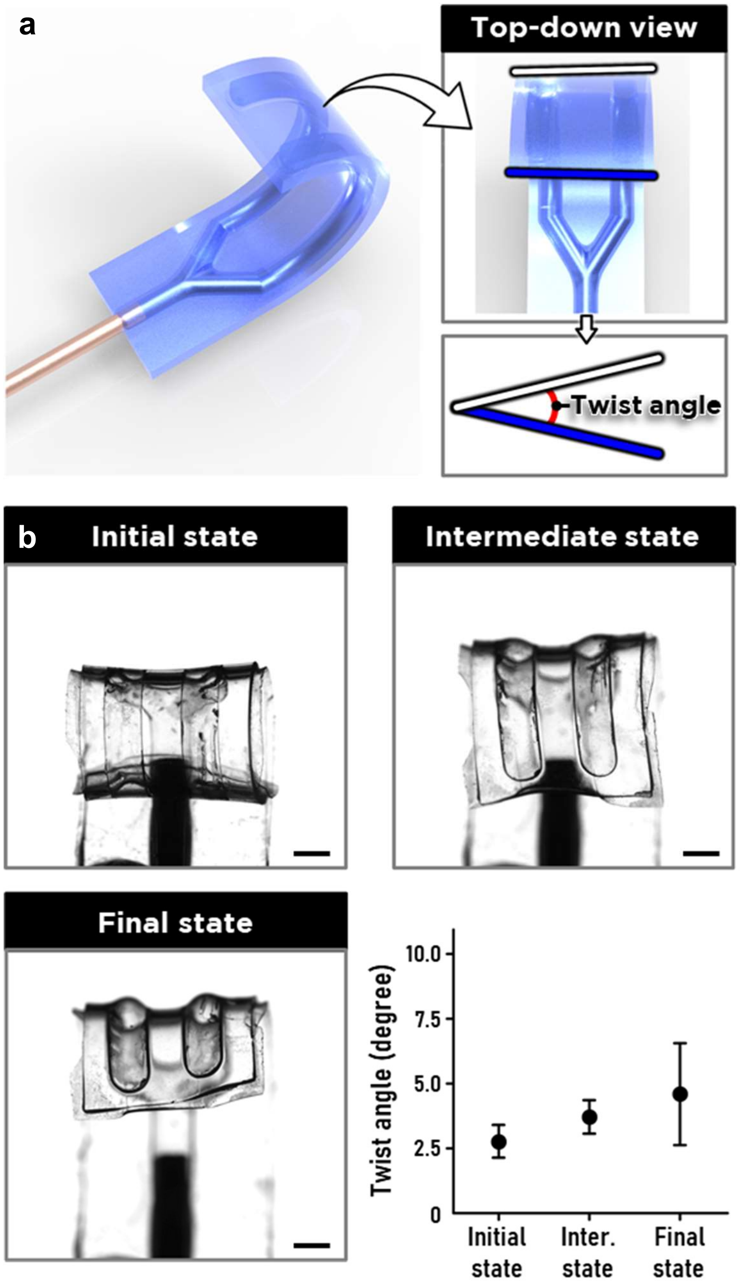

Supplementary Figure 1. Analysis of torsional deformation in the soft-robotic constrictor. **a.** Assessment of torsional deformation using a twist angle defined as the angle between the two ends of the device in the top-down view shown with white and blue lines. **b.** Measurement of the twist angle at three sequential stages of device actuation. No statistical difference in the twist angle indicates the absence of significant torsional deformation of the device. Scale bars, 2mm.

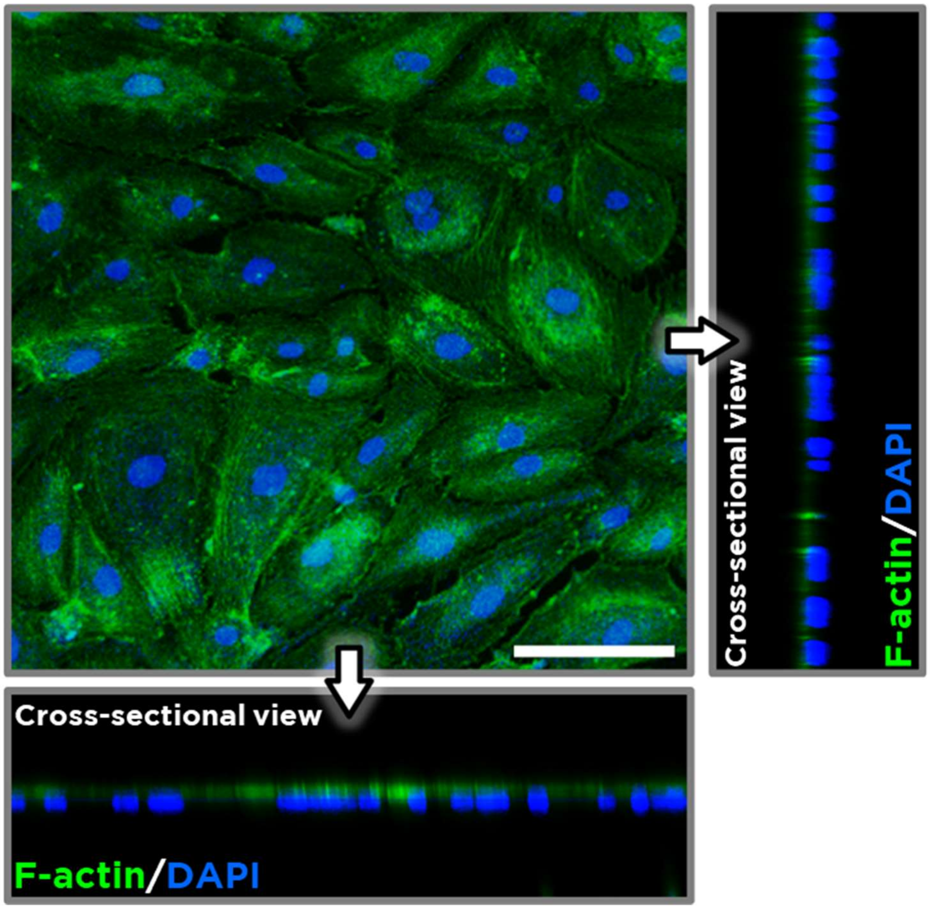

Supplementary Figure 2. Confluent monolayer of HUVECs grown in the soft-robotic actuator. Immunofluorescence staining of cell nuclei clearly shows the formation of a single layer of endothelial cells. Scale bar, 100 μm.

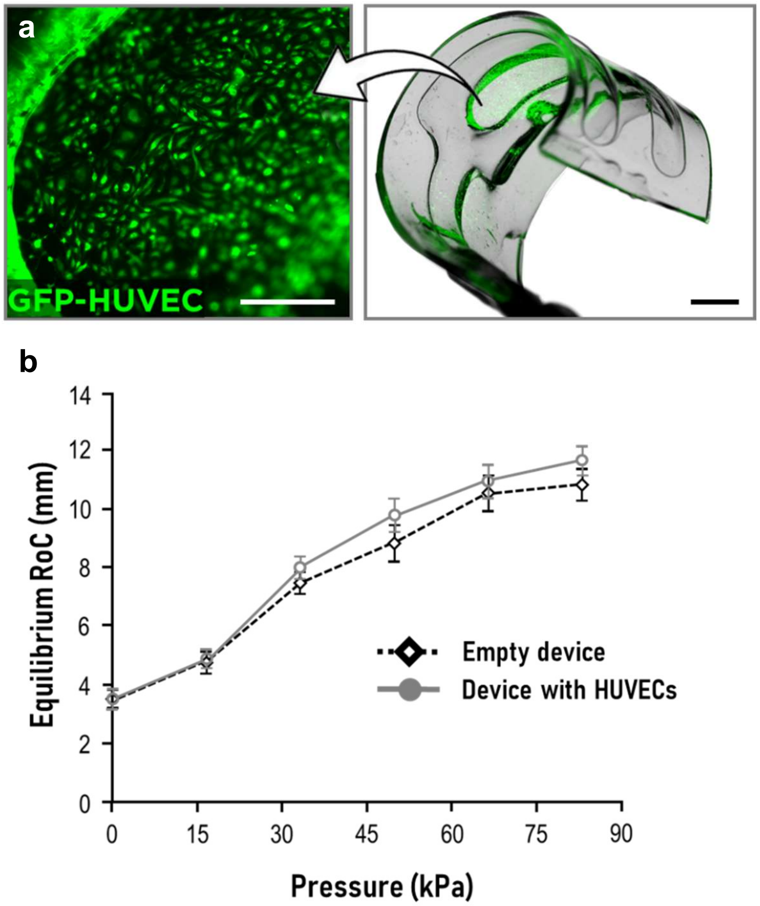

Supplementary Figure 3. Effect of endothelial cell culture on soft-robotic actuation. a. Micrograph of green fluorescence protein (GFP)-expressing HUVECs cultured as a monolayer in the device. Scale bars, 500 µm (left) and 2 mm (right). **b.** Comparison of equilibrium RoC between empty (open diamond) and HUVEC-containing devices (closed circle).

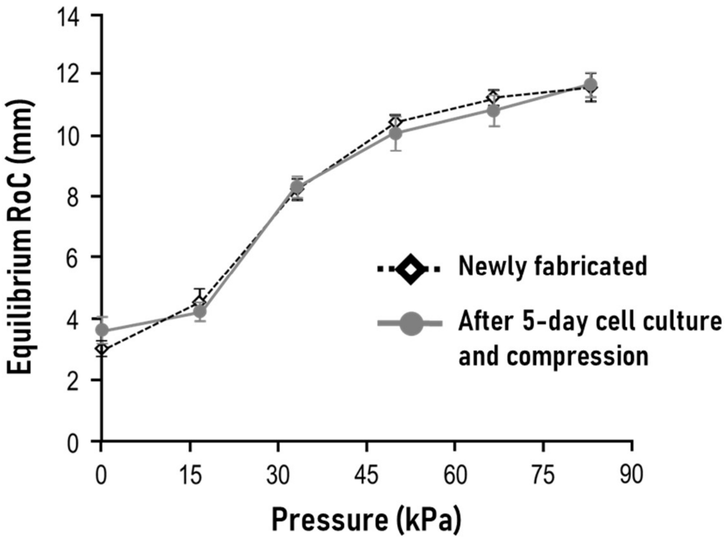

Supplementary Figure 4. Long-term stability of pneumatic actuation in the soft-robotic constrictor. The graph shows comparison of pressure-dependent changes in equilibrium RoC during pneumatic actuation of newly fabricated empty devices (open diamond) and devices in which cells were cultured under dynamic compression for 5 consecutive days and then removed by trypsinization (closed circle).

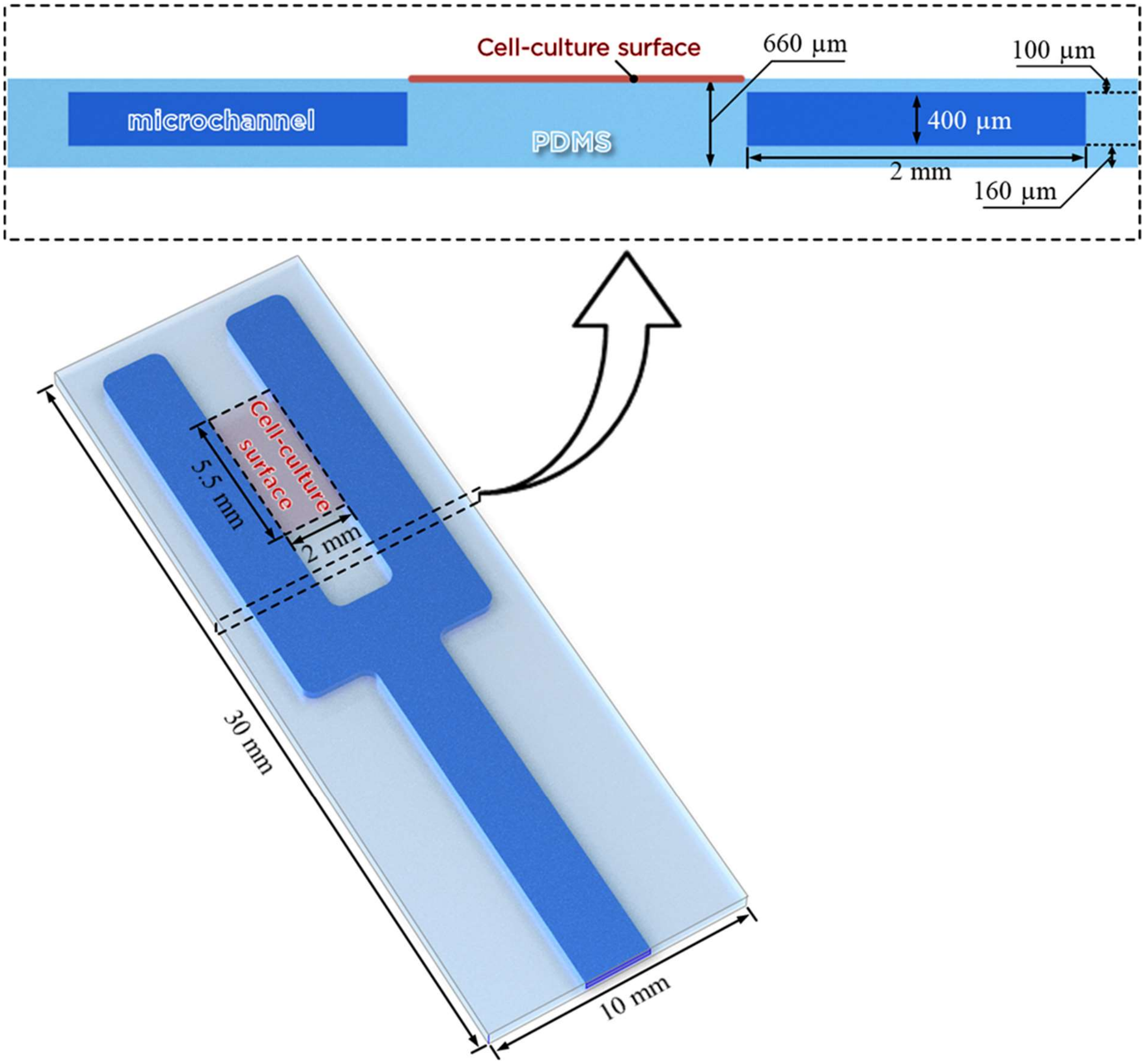

Supplementary Figure 5. Dimensions of the device. The size of the entire device for soft robotic actuation is 10 mm (width) × 30 mm (length) × 0.66 mm (thickness). The device includes two microchannels for injection of compressed air, each of which has the cross-sectional dimensions of 2 mm (width) × 400 μm (height) with top and bottom walls that are 100 μm and 160 μm in thickness, respectively. The cell culture area measuring 2 mm (width) X 5.5 mm (length) is located between the two microchannels.

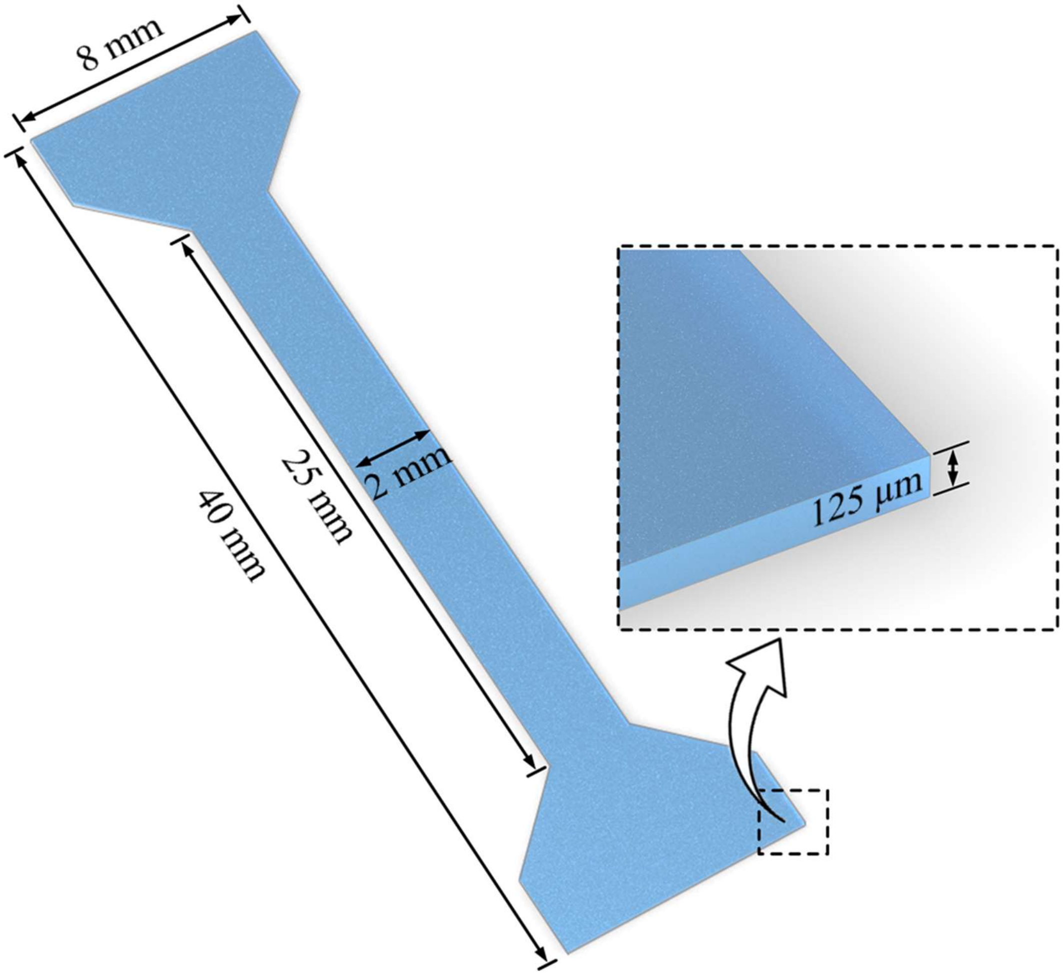

Supplementary Figure 6. Dimensions of the dumbbell-shaped PDMS film. The stretchable dumbbell-shaped PDMS slab is 125 μm-thick and has overall dimensions of 8 mm (width) × 40 mm (length). The main body of the slab is designed to be slender with the size of 2 mm (width) × 25 mm (length) in order to facilitate stretching of the substrate during device fabrication.

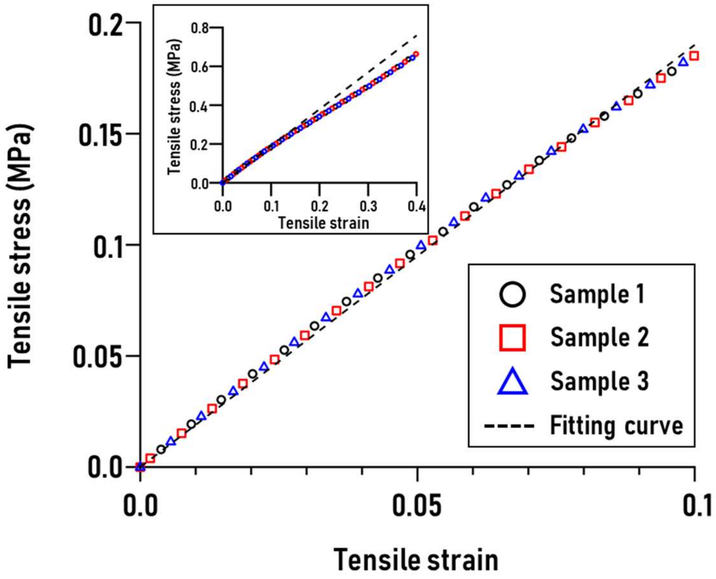

Supplementary Figure 7. Tensile property of PDMS used in the production of the soft-robotic actuator. Stress-strain curves for 3 separate PDMS samples. The graph is shown for tensile strain ranging from 0 to 0.1. The slope of the fitting curve, which represents Young's modulus, is calculated to be 1.9 ± 0.01 MPa. Inset shows stress-strain curves for a full range of strain.

Supplementary Movie 1. This movie shows 3D deformation of the soft robot during a complete cycle of pneumatic actuation (Pressure ON followed by Pressure OFF). The time stamp at the top left corner shows elapsed time in seconds.

Supplementary Movie 2. This movie shows computational simulation of dynamically changing geometry of the soft robot, as well as the level and spatial distribution of maximum nominal principal strain during pneumatic actuation.
